# Analysis of the Efficacy of Acetylcholinesterase Inhibitors in the Treatment of Alzheimer’s Disease, Literature Review

**DOI:** 10.3390/ijms27135733

**Published:** 2026-06-25

**Authors:** Wiktor Petrov, Dawid Ślebioda, Rozalia Kozińska, Klaudia Kukla, Paweł Petrov, Mateusz Sroka, Julia Tesyna, Grzegorz Puźniak, Maciej Kudliński, Tymon Rejda, Izabela Skowron, Agnieszka Chłopaś-Konowałek

**Affiliations:** 1 Scientific Society for Neurotoxicology, Department of Forensic Medicine, Wroclaw Medical University, Mikulicza-Radeckiego 4J, 50-345 Wroclaw, Poland; wiktor.petrov@student.umw.edu.pl (W.P.); dawid.slebioda@student.umw.edu.pl (D.Ś.); klaudia.kukla@student.umw.edu.pl (K.K.); tymon.rejda@student.umw.edu.pl (T.R.); izabela.skowron@student.umw.edu.pl (I.S.); 2 Department of Forensic Medicine, Division of Molecular Techniques, Wroclaw Medical University, Sklodowskiej-Curie 52, 50-369 Wroclaw, Poland

**Keywords:** neurodegenerative diseases, Alzheimer’s disease, acetylcholinesterase inhibitors, tacrine, donepezil, rivastigmine, galantamine, plant extracts

## Abstract

The term ‘dementia’ encompasses a diverse group of progressive neurodegenerative disorders, the common feature of which is the deterioration of higher cortical functions. This process not only involves memory deficits and language communication disorders, but also executive dysfunction and loss of emotional control, which ultimately leads to a complete loss of the patient’s independence. Within this group of disorders, Alzheimer’s disease (AD) presents the most serious clinical challenge, characterized by a unique neuropathological triad: the presence of extracellular β-amyloid plaques, intracellular neurofibrillary tangles of tau protein, and widespread dysfunction of cholinergic transmission. The cholinergic hypothesis remains the cornerstone of the current understanding of cognitive impairment in AD. It posits that progressive dementia is caused by the selective degeneration of neurons in the anterior basal forebrain, resulting in a drastic reduction in acetylcholine (ACh) levels in the synaptic cleft. In the absence of a causal treatment, acetylcholinesterase inhibitors (AChEIs) remain the standard of care. Their pharmacological action is based on the inhibition of the AChE enzyme, which allows neurotransmission deficits to be compensated for by prolonging the half-life of acetylcholine at the synapse. This literature review presents a synthesis of the efficacy and safety of classic and novel AChEIs. A comprehensive search of the PubMed, Scopus, and Cochrane Library databases was conducted for clinical data published up to 2026. Evidence from key trials indicates that standard AChEIs induce significant cognitive stabilization compared to placebo, with rivastigmine maximizing daily living parameters via transdermal delivery. However, their therapeutic impact remains strictly symptomatic without arresting neurodegeneration. Conversely, emerging agents like huperzine A and the translation-blocker Posiphen demonstrate disease-modifying potential by modulating CSF biomarkers associated with amyloid and tau proteins. Clinically, while traditional regimens are limited by gastrointestinal toxicities, transitioning toward innovative multi-target structures represents a necessary shift to address both cognitive decline and neurodegeneration.

## 1. Introduction

### 1.1. Alzheimer’s Disease Within the Spectrum of Dementias: Pathophysiology, Clinical Features, and Diagnostic Challenges

In a more comprehensive sense, dementia is defined as a set of symptoms leading to a decline in cognitive function, particularly memory and executive function [1]. It is estimated that only 2 to 5% of all diagnosed cases of dementia combined are early-onset forms, diagnosed in people under the age of 65 [2]. Alzheimer’s disease (AD) is the most common cause of dementia, currently affecting around 50 million people worldwide. It therefore poses a significant challenge to healthcare systems [1]. This condition is often accompanied by motor and autonomic deficits, which reduce patient survival rates. Due to current demographic trends, it is estimated that by 2050, the global number of dementia cases will increase nearly threefold, and in Europe alone, it will double [3]. AD is underpinned by a progressive neurodegenerative process and the accumulation of pathological protein structures, such as beta-amyloid plaques and hyperphosphorylated tau protein [1]. This pathology develops insidiously—neuronal loss and synaptic dysfunction may occur even a dozen or so years before the first observable deficits become apparent [4]. AD manifests itself through a range of distressing non-cognitive symptoms. The most common include akathisia, perseverative behaviors, and various forms of irritability or even aggression. This results not only in a decline in intellectual ability and changes in behavior, but above all, in the gradual loss of the patient’s ability to function independently in daily life and an intensification of psychoneurological symptoms [5]. Patients also exhibit states of apathy interspersed with periods of severe irritability, which consequently places a burden on the lives of their loved ones and leads to the disorganization of the social aspect of their lives [6]. It is noteworthy that nowadays we can detect and expand our understanding about other conditions related to dementia such as limbic-dominant TDP-43 encephalopathy [2]. The process of neurodegeneration is difficult to detect in its early stages, as this preclinical phase can last for years without manifesting any clear external signs. Although traditional diagnostics often rely on the analysis of individual indicators, combining specific markers such as P-tau or Aβ1-42 presents better results. The use of P-Tau alone allows for a better distinction between AD and other types of dementia, whereas the inclusion of Aβ1-42 in the tests increases the effectiveness of predicting the development of the given condition [7].

### 1.2. The Role of Acetylcholine in the Nervous System

Acetylcholine (ACh) is a neurotransmitter fulfilling various roles in the peripheral nervous system (PNS) and central nervous system (CNS). ACh is an endogenous compound, an ester consisting of choline and acetic acid [8]. ACh is responsible for both essential functions and higher functions, such as attention and memory. Acetylcholine modulates synaptic plasticity. Cholinergic systems modulate inflammation and neurotransmitter release, which greatly influence higher cognitive functions. Moreover, acetylcholine modulates the action of inhibitory and excitatory synapses in the hippocampus [9]. Adequate levels of acetylcholine are necessary to maintain cognitive functions; therefore, the cholinergic hypothesis gained recognition among scientists studying the pathophysiology of AD. A broad knowledge of the physiology and pathophysiology of the cholinergic system is necessary to understand the molecular mechanisms underlying AD and to develop appropriate treatment.

### 1.3. A Brief Overview of Cholinergic System Physiology

There are three types of cholinergic neurons: motor neurons, interneurons, and projection neurons. This publication will focus on the role of the latter two. Cholinergic projection neurons and interneurons are located across the cortex, brainstem, and the base of the forebrain. Neurons located in the forebrain are known as basal forebrain cholinergic neurons, which are especially prominent in the medial septum, vertical and horizontal subdivisions of the diagonal band, the ventral pallidum, the substantia innominata, and the nucleus basalis. In the cortex, cholinergic neurons have rostromedially and caudolateral projections: rostrally located cholinergic neurons project to medial neocortical regions, whereas caudally located cholinergic neurons project to lateral neocortical regions [10]. Laminar selectivity characterizes cholinergic neurons. Basal forebrain cholinergic neurons are characterized by great heterogeneity, introducing further ambiguities in AD research. Neurotransmission is impossible without receptors. Receptors act as neuromodulators; they regulate the release of neurotransmitters, and consequently, they are in control of the rate and probability of synaptic transmission. Cholinergic receptors are widespread across the somatic and autonomic nervous system. In cholinergic systems, there are two types of receptors: muscarinic and nicotinic (nAChRs) [11]. Nicotinic receptors are traditionally divided into a muscle type, present in the PNS on skeletal muscles at neuromuscular junctions, and a neuronal type, present in autonomic ganglia and widely distributed throughout the CNS. This older functional classification (previously denoted N1 and N2) has largely been replaced by a subunit-based nomenclature; among the neuronal receptors, the α7 and α4β2 subtypes are the most abundant in the brain and the most relevant to AD. Nicotinic receptors are pentameric, non-selective, ligand-gated cation channels that comprise a combination of 17 subunits (α1-α10, β1-β4, γ, δ, and ε) gathered around a single pore. Those seventeen subunits have a limited number of pentameric combinations, different for muscle-type and neuronal-type receptors [11]. All nAChRs are similar in structure, with a large N-terminal domain facilitating the binding of ligands. Furthermore, nAChRs have four hydrophobic transmembrane regions (M1, M2, M3, M4) that differ structurally from the N-terminal domain. The short extracellular C-terminus and large intracellular loop are the other two components. In general, there are two types of nicotinic acetylcholine receptors: heteromeric and homomeric. The unique structure of nicotinic acetylcholine receptors enables three conformational states: closed channel, which corresponds to a resting state; an open activated state that requires stabilization by the positive allosteric modulator and the agonist epibatidine; and the last state is desensitized, where the ion channel is closed [11]. The nAChRs are part of the cys-loop receptor family; these receptors can be activated by endogenous ligands—ACh or other exogenous ligands.

### 1.4. The Cholinergic Hypothesis of Alzheimer’s Disease

The cholinergic hypothesis of AD gained traction in the second half of the twentieth century. This theory was developed as a result of the observation that acetylcholine levels in patients with AD are significantly lower than in healthy individuals. AD is characterized by substantial cholinergic deficits and the degeneration of cholinergic neurons, demonstrated by a loss of cholinergic markers in the cortex. The loss of these markers correlates with the progression of AD symptoms. There is a correlation between the number of basal forebrain cholinergic neurons and cognitive decline. Levels of ACh are maintained by choline acetyltransferase (ChAT) and AChE [12,13]. Rather than impairing the breakdown of ACh, AD causes the degeneration of cholinergic neurons and a consequent reduction in cholinergic transmission and acetylcholine availability. It is hypothesized that decreased levels of acetylcholine make the brain tissue more susceptible to the aggregation of β-amyloid plaques and tau tangles. It is widely suspected that the relationship between ACh levels and the amount of β-amyloid plaques is bidirectional. Increasing acetylcholine levels has therapeutic benefits, alleviating symptoms related to memory loss and agitation. One of the major drawbacks of acetylcholine-based treatment is that it does not stop the progression of the disease. Functional improvement is well-demonstrated by improved scores on neuropsychiatric tests [14].

### 1.5. Pharmacological and Non-Pharmacological Management of Alzheimer’s Disease

AD is a progressive neurodegenerative disorder that currently has no cure. However, the rate of disease progression can be significantly reduced. Effective management of AD requires a comprehensive approach that integrates pharmacological treatments with non-pharmacological interventions to address the complex cognitive, functional, and behavioral needs of patients. Non-pharmacological interventions are recommended as adjuncts to pharmacotherapy [15]. Specifically, cognitive training has been shown to enhance working memory, verbal memory, verbal fluency, confrontation naming, and attention [16]. In the early stages of AD, when mild cognitive impairment (MCI) is present, lifestyle and dietary modifications, such as increased physical activity and adherence to a Mediterranean diet, have been effective in reducing symptoms and significantly improving AD biomarkers [17,18,19]. Pharmacological treatments for AD are categorized as either symptom-mitigating or disease-modifying drugs. The choice of intervention depends on disease severity. The primary objectives of pharmacological therapy are to alleviate symptoms, slow disease progression, and enhance patients’ quality of life [20]. Disease-modifying drugs, such as monoclonal antibodies targeting amyloid-β plaques, include lecanemab and donanemab, which are indicated for the MCI and mild dementia stages of AD; aducanumab, the first agent in this class, received accelerated approval in 2021 but was withdrawn from the market by its manufacturer in 2024 [20]. Symptom-mitigating drugs consist of acetylcholinesterase inhibitors and glutamate modulators. Memantine, the only FDA-approved glutamate modulator, is prescribed for moderate to severe AD. FDA-approved acetylcholinesterase inhibitors, including donepezil, galantamine, and rivastigmine, are used to treat mild-to-moderate stages of the disease [20]. With the introduction of these anti-amyloid monoclonal antibodies and biomarker-guided treatment, acetylcholinesterase inhibitors are no longer the only disease-relevant option; nevertheless, they remain central to the symptomatic management of mild-to-moderate AD. Beyond this, other co-occurring neuropsychiatric symptoms can be treated. In the treatment of agitation, brexpiprazole can be used due to its high effectiveness and low side-effect profile [21]. The risk of extrapyramidal symptoms and somnolence, associated with other typical and atypical neuroleptics, significantly outweighs the benefits [21,22]. In the case of psychosis, neuroleptics should be used with caution, as clinical studies have demonstrated the increased risk of death compared to placebo [23]. Selective serotonin reuptake inhibitors are safe and effective in the treatment of depression symptoms associated with AD [24]. Sleep disturbances pose a significant issue to AD patients, as insomnia can exacerbate cognitive decline [25]. Melatonin, trazodone, orexin antagonists, and acetylcholinesterase inhibitors are used in order to improve various aspects of sleep architecture and circadian rhythm [25,26].

This narrative review aims to provide a comprehensive, up-to-date overview of the current knowledge on the clinical efficacy, pharmacological properties, and safety profiles of selected acetylcholinesterase inhibitors for the treatment of neurodegenerative diseases, with particular emphasis on AD. The specific objective of this study was to analyze and compare the effectiveness of donepezil, rivastigmine, and galantamine in improving cognitive function, daily functioning, and global clinical outcomes in patients with AD, based on available clinical trial data. Tacrine, memantine, and selected emerging and plant-derived compounds are also discussed in order to provide historical and translational context and to clarify the broader scope of the review. By synthesizing data from cohort studies, randomized controlled trials, and meta-analyses, this review seeks to identify patterns of therapeutic response, highlight differences between individual agents, and indicate potential directions for future research.

## 2. Methodology

This article was designed as a structured narrative literature review of acetylcholinesterase inhibitors (AChEIs) in Alzheimer’s disease (AD), with an emphasis on pharmacological characteristics, clinical efficacy, safety, and tolerability. The review was not intended as a systematic review or meta-analysis. However, to improve transparency, the literature search strategy, eligibility criteria, study selection approach, evidence prioritization, and methodological limitations are described below.

A literature search was conducted in PubMed, Scopus, and Google Scholar. The search covered publications from February 1981 to August 2025. The following keywords and keyword combinations were used: “Alzheimer’s disease”, “Alzheimer-type dementia”, “acetylcholinesterase inhibitors”, “AChEI”, “cholinesterase inhibitor”, “acetylcholinesterase inhibition”, “butyrylcholinesterase”, “butyrylcholinesterase inhibitors”, “cholinergic hypothesis”, “tacrine”, “donepezil”, “rivastigmine”, “galantamine”, “memantine”, “efficacy”, “effectiveness”, “safety”, “adverse effects”, “side effects”, “hepatotoxicity”, “pharmacokinetics”, “bioavailability”, “blood–brain barrier,” “huperzine A”, “posiphen”, “phenserine”, “plant extracts”, “herbal medicine”, “phytotherapy”, and “alkaloids”.

Studies were considered eligible for inclusion if they were published between February 1981 and August 2025, were available in English or Polish, had accessible full text, and included clinically or pharmacologically relevant data concerning AChEIs, particularly donepezil, rivastigmine, and galantamine. Eligible publication types included randomized controlled trials, non-randomized clinical studies, observational studies, systematic reviews, meta-analyses, narrative reviews, pharmacological studies, preclinical studies, and clinically relevant guideline or regulatory documents.

Publications were included if they addressed at least one of the following areas: pharmacodynamic or pharmacokinetic properties of AChEIs; cholinergic dysfunction in AD; clinical efficacy or symptomatic effects of approved AChEIs; safety, tolerability, and adverse effects; comparative or indirect evidence concerning approved AChEIs; or contextual information on tacrine, memantine, huperzine A, posiphen, phenserine, plant-derived compounds, or AChEI use in other neurodegenerative disorders.

Publications were excluded if they were not directly related to cholinesterase inhibition, AD, dementia, or neurodegenerative disorders; if they were not published in English or Polish; or if they did not contribute relevant pharmacological, efficacy, safety, or tolerability information. Conference abstracts, letters, editorials, and commentaries were generally excluded unless they provided clinically important contextual information.

Study selection was based on relevance to the review objectives and was performed through title and abstract screening, followed by full-text assessment of potentially relevant publications. For the discussion of clinical efficacy and safety of approved AChEIs, priority was given to randomized controlled trials, systematic reviews, and meta-analyses. Observational studies, narrative reviews, preclinical studies, and older foundational publications were included when they provided relevant mechanistic, historical, pharmacological, or clinical context. A total of 111 publications were included in the final narrative synthesis.

Because this was a structured narrative review rather than a systematic review, no formal review protocol, PRISMA flow diagram, comprehensive record-count reporting, meta-analysis, or risk-of-bias assessment for each included study was performed. Data were synthesized narratively, with emphasis on mechanistic coherence, clinical relevance, consistency of findings across studies, and limitations arising from heterogeneity in study design, disease stage, population characteristics, drug dose, outcome, and treatment duration. Therefore, conclusions regarding comparative efficacy between donepezil, rivastigmine, and galantamine should be interpreted cautiously. The review aims to summarize and contextualize the available evidence rather than establish definitive superiority of one agent over another.

## 3. Characteristics of AChEIs Used in the Symptomatic Treatment of Alzheimer’s Disease

Despite the fact that AD was first described in 1907, its therapeutic options remain quite limited [27]. Presently, only four drugs are available: three AChEIs (donepezil, rivastigmine, and galantamine) and the N-methyl-D-aspartate (NMDA) receptor antagonist memantine [28,29,30,31,32,33]. Unfortunately, none of these drugs affect the cause of the disease—they can only alleviate its symptoms [28,30,33,34]. Research on AD has led to the so-called cholinergic hypothesis, according to which there is a deficiency of ACh in the CNS during the course of the disease [28,32,35]. This hypothesis is described in detail in Section 1.4: The cholinergic hypothesis of AD. This knowledge constituted the foundation for the development of AChEI drugs, which are designed to inhibit the activity of the enzyme AChE, which is responsible for the degradation of ACh at the synaptic cleft [28,29,32,34,35]. Increased cholinergic neurotransmission has been observed to enhance cognitive function and daily functioning in patients, without exerting an influence on the progression of the underlying disease [29,34,36]. In this publication, we focus on describing all clinically used AChEIs, beginning with tacrine, which, as the first drug employed in the pharmacotherapy of AD, constitutes a historical and therapeutic reference point for the development and application of subsequent agents in this class. The approval of tacrine for clinical use in 1993 marked a breakthrough in AD therapy and served as a driving force for the development of later drugs, while both its beneficial effects and adverse reactions became the basis for creating newer, more effective, and safer preparations.

### 3.1. Tacrine

#### 3.1.1. Characteristics of Tacrine

**Figure 1:**
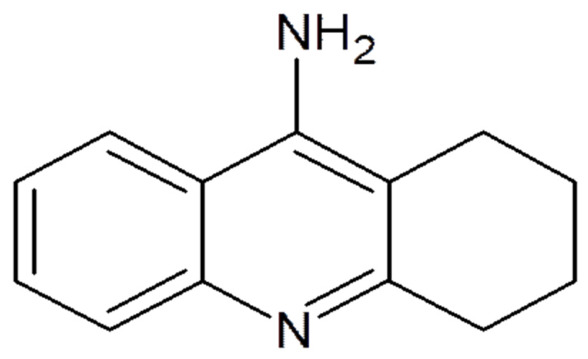
Structural formulas of tacrine (prepared using ChemSketch Version 12.01).

**Figure 2:**
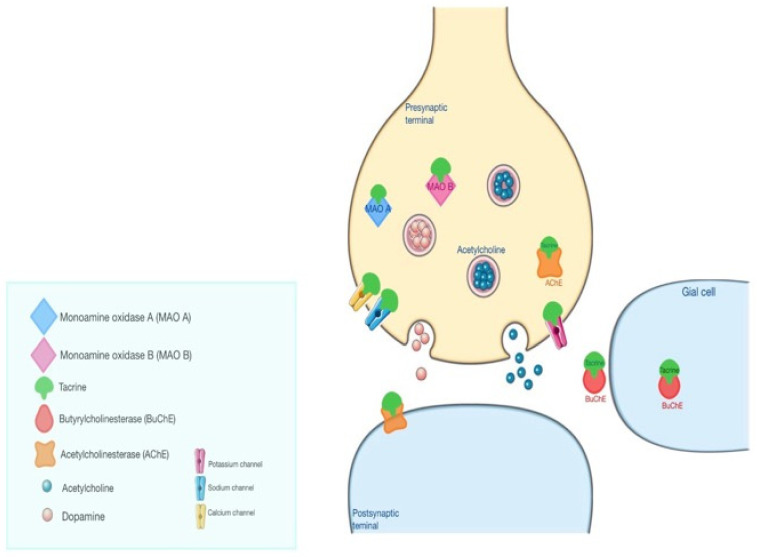
Molecular mechanism of tacrine. Tacrine reversibly and non-competitively inhibits AChE and BuChE, resulting in an increased concentration of ACh at synapses. In addition, tacrine blocks the potassium, sodium, and calcium channels. The substance reduces MAO-A and MAO-B activity, which results in an increased concentration of dopamine. Adapted from Servier Medical Art (https://smart.servier.com [accessed on 28 May 2026]), licensed under CC BY 4.0 (https://creativecommons.org/licenses/by/4.0/ [accessed on 28 May 2026]).

The compound 1,2,3,4-tetrahydroacridine-9-amine, later known as tacrine (Figure 1), was first synthesized during World War II [35]. Concurrently, a team under the direction of Adrien Albert sought an intravenous antiseptic that would be safe for use in treating the wounds of soldiers. Scientists synthesized a total of 90 monoaminoacridines, several of which exhibited effects on the CNS. However, among these compounds, tacrine emerged as a particularly noteworthy contender [35,36]. In the 1950s, it was utilized for various applications, including the reversal of the effects of chloroform- and morphine-induced anesthesia in dogs [37]. Initially, it was used to treat psychosis or comas resulting from drug overdose. Subsequent studies demonstrated that the observed CNS stimulant effects were attributable to the inhibition of AChE and butyrylcholinesterase (BChE) (Figure 2) [37,38]. In 1976, a central cholinergic deficit was identified in patients diagnosed with AD [35]. This event initiated the utilization of tacrine, a pharmaceutical agent, to elevate ACh levels within the brain. In the 1980s, a pilot study was conducted with tacrine administered intravenously, which showed improvement in 9 out of 12 patients [39,40]. Subsequent studies have demonstrated a moderate efficacy of tacrine in improving cognitive function. Patients demonstrated enhanced performance on the Mini-Mental State Examination (MMSE) and AD Assessment Scale-Cognitive Section (ADAS-Cog) scales. However, these improvements did not translate into meaningful benefits in their daily functioning. Nonetheless, these enhancements did result in a delay in the necessity of nursing home admission. A significant number of tacrine discontinuations have been documented in numerous studies due to adverse effects [36,40,41].

In September 1993, tacrine became the first drug approved by the FDA for the treatment of AD [28,35,37,42]. Tacrine exerts its effects by reversibly and non-competitively inhibiting AChE and BChE. Consequently, this results in an increased concentration of ACh at synapses and an improvement of cognitive function [32,35,38,43,44]. Additionally, the substance exhibits multiple mechanisms of action, including the blocking of potassium, sodium, and calcium channels; reduction in monoamine oxidase A (MAO-A) and B (MAO-B) activity; inhibition of 5-hydroxytryptamine receptors and dopamine reuptake; interaction with muscarinic receptors; increase in the number of nicotinic receptors; and a role as a weak NMDA receptor agonist [33,38,45].

#### 3.1.2. Pharmacokinetics of Tacrine

The therapeutic benefits of tacrine are primarily determined by its bioavailability, which depends on the route of administration, dosage, individual absorption capacity, and hepatic metabolic rate. Tacrine demonstrates rapid absorption; however, its bioavailability is limited to 10–30%. The concomitant ingestion of a meal with the drug has been shown to reduce its absorption by as much as 40% [38,40,45,46]. Tacrine has been found to bind to plasma proteins in 55% of cases, and its volume of distribution has been determined to be 182 L. The drug reaches its maximum plasma concentration (Cmax) after approximately two hours, and the half-life of a single dose is estimated to be between three and six hours. In the final phase of elimination, the average half-life is about 2.5–4 h [38,44,45,46]. The medication attains a steady state after a 24-h period of administration, with a frequency of four times per day [45]. Tacrine has been observed to penetrate the blood–brain barrier [35,37]. It undergoes a first-pass effect and is metabolized by cytochrome P450, which is most likely responsible for its toxicity to the liver [35,43,44]. The oral formulation of tacrine was marketed in the form of capsules, with each capsule containing a specific dose of the medication. In the studies of Samuels et al. [40] and Marucci et al. [47], tacrine was used in doses of 10, 20, 30, and 40 milligrams. In these studies, the dosage was initiated at 40 mg per day and subsequently augmented to the highest tolerable dose, ranging from 160–200 mg. The medication was used for cases of mild to moderate AD [43,46].

#### 3.1.3. Safety of Tacrine

The adverse effects of tacrine can be categorized into two distinct groups: cholinergic and idiosyncratic. Typical cholinergic effects resulting from increased levels of ACh in the PNS include nausea, vomiting, lack of appetite, diarrhea, increased gastric acid secretion, bronchial secretion, abdominal pain, salivation, excessive sweating, lacrimation, polyuria, myalgia, rhinitis, sleep disturbances, weight loss, and autonomic nervous system disorders (in approximately 48% of patients). The administration of tacrine with a meal has been demonstrated to result in a partial alleviation of gastrointestinal symptoms. However, this also reduces the agent’s bioavailability [32,40,47,48,49,50]. The most concerning findings were the liver abnormalities, which manifested as elevated aminotransferases [43,44,49,50]. While these alterations generally manifested asymptomatically, continuous monitoring was necessary to ensure appropriate management. The FDA recommended weekly monitoring of AST and ALT [35,40,44]. According to the findings of several studies, there was an increase in transaminase levels observed in 25–50% of patients [36,40,44,46,47,51,52]. The presence of ALT > 3 × ULN was observed in 25% of the cohort, while ALT > 20 × ULN was detected in 2%. In certain cases, levels of ALT > 50 × ULN were identified. The aforementioned alterations typically manifested within 6 to 12 weeks following the initiation of therapy and were resolved up to 6 weeks after treatment discontinuation [40,44,47,48,49]. In 88% of cases, treatment could be resumed without the recurrence of adverse effects [40,53]. In rare instances, elevations in alkaline phosphatase, bilirubin, or jaundice have been documented. The following have also been reported: seizures, rash (1–2%), and dizziness [48,49]. The decision to remove tacrine from the market was precipitated by several factors, including its hepatotoxicity, the inconvenient dosing regimen (requiring administration four times a day), and the emergence of newer AChEIs that demonstrated a superior safety profile. This decision was formally communicated by the FDA in 2013 [32,38,43,47]. To this day, the compound remains under study as a potential basis for developing new drugs against AD. It can be used as a foundation for the synthesis of new compounds or as a component of hybrid multitarget drugs that may transform future approaches to AD treatment [29,33,38,43].

### 3.2. Donepezil

#### 3.2.1. Characteristics of Donepezil

**Figure 3:**
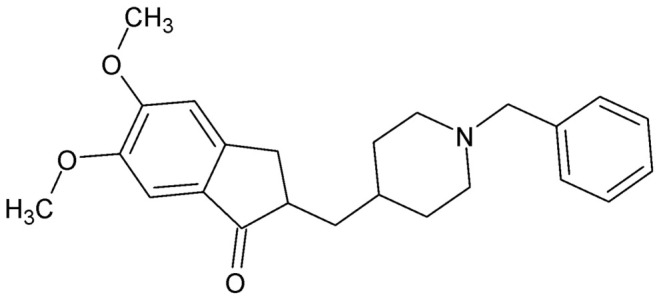
Structural formulas of donepezil (prepared using ChemSketch).

Donepezil, sold under the brand name Aricept^®^, is the compound (RS)-2-[(1-benzyl piperydyn-4-ylo)metylo]-5,6-dimetoksy-2,3-dihydroinden-1-on, as shown in Figure 3. Donepezil was the second drug approved by the FDA in 1996 for the treatment of mild to moderate AD [54].

It is recommended that the initial dose be in the range of 5 milligrams per day for 4–6 weeks, then the dose should be increased to 10 milligrams per day, and then after 3 months, the daily dosage should reach 23 milligrams for the treatment of moderate to severe forms of the disease [55]. The pharmaceutical agent under consideration is a cholinergic drug, which is classified as an AChEI. It exerts its effects on this enzyme by binding to its anionic site in a central location. The binding is reversible and non-competitive. Consequently, the action of AChE is blocked, preventing the hydrolysis of ACh. Donepezil hydrochloride contains a benzylpiperidine moiety bound by a methyl group to dimethoxyindanone. Due to its unique molecular structure, the substance has the capacity to inhibit both peripheral and active anionic sites simultaneously [56]. Donepezil exhibits a 1250-fold higher affinity for AChE than for BChE, thereby demonstrating selective affinity at this level [54]. This characteristic is pertinent to clinical applications in the treatment of AD. This effect is attributable to the replenishment of the neurotransmitter’s deficiency. However, it is noteworthy that the class of AChEIs merely postpones the progression of AD pathogenesis, without achieving its complete eradication. Donepezil exerts its effects at the level of neurotransmission, and moreover, at the cellular and molecular levels. These effects occur at all stages of the pathogenesis of AD, and they include a decrease in the cytotoxic effect of glutamate and an increase in the neuroprotective isoform of AChE [32,57]. This finding is particularly noteworthy in light of recent theories concerning the etiology of disease, which attribute a significant role to Aβ and Tau. This underscores the substantial impact of neurotoxic factors, oxidative stress, iron accumulation, and cholesterol deposition on neuronal connections, leading to a disruption of the signaling cascade [58]. In reference to the aforementioned hypothesis, novel hybrid forms of donepezil have begun to be developed. These new forms have the potential to exhibit both good neurotransmission and neuroprotection effects. This is due to their ability to effectively inhibit the escalation of oxidative stress and the deposition of tau protein. The development of hybrid compounds has led to the creation of donepezil-AP2238 and donepezil-tacrine. The former was selected as the matrix for a series of donepezil-AP2238 hybrids due to its effective antitoxic activity. The donepezil-tacrine analogue has been observed to inhibit AChE with greater efficacy and concurrently demonstrate substantial anti-aggregation activity [32].

#### 3.2.2. Efficacy of Donepezil

As presented by Boada-Rovira et al. in 2004 [59], donepezil treatment resulted in statistically significant improvements in patients’ cognitive function, activity, and social behavior. The treatment was well-tolerated, despite high levels of comorbidities (at the beginning in 745 of the participants) and concomitant medication use (802 of them was treated with at least one other medication). In their research, 1113 patients were treated with donepezil, but 59 of them did not reach the endline due to adverse effects.

The treatment effect was observed in both mild and moderate cohorts. Patients were stratified by severity. However, a direct comparison of results with data from other studies, especially those without a control group, is inappropriate [59]. Baseline dementia severity significantly influenced the effect of donepezil on standardized MMSE scores, with greater benefits observed in patients with moderate disease (MMSE scores 10–13) than in patients with severe disease (MMSE scores 5–9). The mean difference in scores between the groups continuing donepezil and those discontinuing donepezil was 2.6 points (95% CI, 1.5–3.7) in patients with moderate disease [60].

#### 3.2.3. Pharmacokinetics of Donepezil

The Cmax was found to be 3.2, 7.0, and 12.1 μg/L, achieved after doses of 2, 4, and 6 mg at approximately 4 h. It has been observed that with a 2 mg dose of donepezil, absorption was not affected by consuming a meal 30 min before taking the drug [61]. Metabolized by cytochrome P450 isoenzymes 3A4 and 2D6, it undergoes extensive first-pass metabolism. The main metabolites are a hydrolysis product, two glucuronide conjugates, and an oxidation product. One of these metabolites is active and exhibits activity similar to the parent drug. However, donepezil and its metabolites are eliminated primarily via the renal route [62]. In elderly patients receiving single doses of donepezil 2 mg, time to maximum concertation (Tmax), mean retention time, and T_1/2_ were longer, and Vd was significantly longer than in young subjects. Renal or hepatic impairment did not affect the pharmacokinetic parameters of donepezil [62].

#### 3.2.4. Safety of Donepezil

Coadministration of donepezil 5 mg with cimetidine, digoxin, theophylline, or warfarin was not associated with any clinically significant changes (>20% change) in the kinetics of either donepezil or the concurrent agents [63]. At doses of 5 and 10 mg of donepezil, the heart rate decreased but was not considered clinically significant, and the incidence of bradycardia with donepezil was not significantly different from that observed with the placebo [62]. The most common adverse reactions reported in clinical trials were gastrointestinal events, typically nausea, vomiting, diarrhea, and constipation. Headache, dizziness, and sleep disturbances have also been reported [64].

### 3.3. Rivastigmine

#### 3.3.1. Characteristics of Rivastigmine

**Figure 4:**
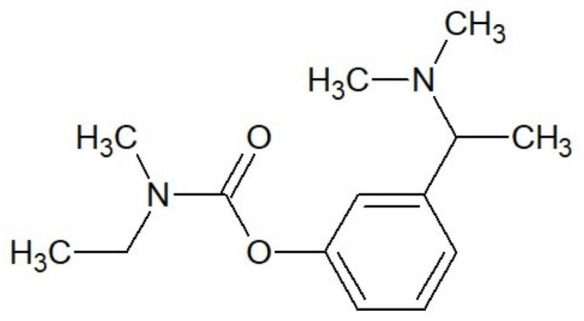
Structural formulas of rivastigmine (prepared using ChemSketch).

Rivastigmine (N-ethyl-N-methylcarbamate of (S)-3-[1-(dimethylamino)ethyl]phenyl), marked under trade names as Atmina, Evertas, Exelon, Nimvastid, Ristidic, and Rivastigmin NeuroPharma, is an organic chemical compound belonging to the carbamate class (Figure 4).

The drug exhibits relatively low binding to proteins and reduced capacity for interaction with other drugs [54]. These characteristics serve to distinguish it from other AChEIs. Rivastigmine is an oral preparation used for the symptomatic treatment of mild to moderate AD. The product received official approval and marketing authorization from the FDA in 2000. Cholinergic neuronal degeneration and reduced cholinergic transmission, including a significantly reduced number of nicotinic receptors, are responsible for the decline in cognitive function in AD [65]. Rivastigmine, a parasympathomimetic agent, has been shown to mitigate this deficit by impeding ACh degradation through the inhibition of the enzymes responsible for this process [30]. Specifically, it functions as a dual AChEI, targeting the G1 isoform, and BChE. These enzymes play a crucial role in the regulation of parasympathetic excitation [20]. The inhibition of cholinesterases by rivastigmine is a result of its binding to the anionic and esteratic sites of these enzymes. This binding mechanism results in the inactivation of the cholinesterases and the subsequent prevention of their binding to the neurotransmitter—acetylcholine [66]. However, this mechanism merely contributes to the symptomatic treatment of AD, without preventing its progression.

#### 3.3.2. Efficacy of Rivastigmine

In the context of AD, rivastigmine and donepezil exhibit different pharmacological profiles regarding their interaction with cholinesterases. Specifically, donepezil functions as a selective AChEI [56], maintaining its independence from the regulation of BChE levels. This pharmacological distinction is of particular significance given the observed changes in enzyme levels during disease progression: AChE levels may decrease by up to 90% in specific regions of the CNS, while BChE levels have been shown to increase, reaching values up to 120% of the physiological state [67]. One study indicated an increase in the BuChE/AChE ratio, reaching up to 165% in some cases [54]. This phenomenon occurs in parallel with the progression of AD. Consequently, the BuChE/AChE ratio has been observed to shift from a pre-disease value of 0.2 before the disease to a value of 11 [68]. Consequently, BChE may play a more substantial role in the hydrolysis of a considerable amount of ACh in the CNS affected by AD [69].

#### 3.3.3. Pharmacokinetics of Rivastigmine

Rivastigmine is absorbed after oral administration, with a mean absorption time of 0.8 to 167 h and an efficacy of 90%. However, the rate of absorption is influenced by the ingestion of food, with the absorption rate reduced by approximately 30% when food is consumed prior to rivastigmine administration. Notably, it does not undergo a first-pass effect in the liver; rather, it is converted directly in the CNS. Subsequently, it is transported to the hepatic bloodstream, where it undergoes N-demethylation. However, its metabolism by CYP450 is minimal due to its relatively weak binding to proteins. Consequently, rivastigmine therapy is not anticipated to exhibit a high degree of interaction with other pharmaceutical agents. This is a salient consideration for a substance primarily intended for elderly individuals, who frequently utilize multiple medications, both for primary conditions and to treat comorbidities. The half-life of the substance has been shown to range up to 2 h [70]. An additional effect of rivastigmine is its ability to increase the levels of P-glycoprotein as well as receptor-conjugated protein for lipoprotein 1. Lower levels of these proteins have been observed in the aging population as well as in age-related conditions, which may result in increased amounts of Aβ in the brain [71]. Rivastigmine is distinct from other AChEIs in that it is available in two different formulations for patient administration. While other AChEIs are typically available in oral preparations, rivastigmine can be administered either as a capsule (dosages ranging from 1.5 to 6 milligrams) or as a transdermal system (with varying rates of drug release, ranging from 4.6 to 17.4 milligrams per 24 h) [20]. The introduction of this route of administration of rivastigmine was made possible by the drug’s properties, including its small particle size (approximately 250 Da) and its lipo- and hydrophilicity [72]. The transdermal patch offers unique formulation advantages and may be more conducive to patient adherence to therapeutic recommendations [73]. Furthermore, the active substance’s direct absorption into the bloodstream through the skin enables it to circumvent the gastrointestinal tract. This process bypasses the first-pass effects and hepatic biotransformation, thus ensuring a more favorable pharmacodynamics profile. Therefore, the effects of a 9.5 mg/24 h patch dose are comparable to those provided by 12 mg rivastigmine capsules per day [74], with a significantly lower Cmax (Cmax: 8.7 vs. 21.6 ng/mL) and a longer time after which maximum drug concentration is reached (8.1 vs. 1.4 h) [72].

#### 3.3.4. Safety of Rivastigmine

Rivastigmine, like other AChEIs, is associated with a range of adverse effects that stem from excessive PNS. While its pharmacological profile is reported to potentially offer a reduction in peripheral side effects compared with some other AChEIs [75], the observed toxicity of this drug is dose-dependent, suggesting that increasing the dosage may result in an escalation of adverse effects. The occurrence of these events is primarily observed in the gastrointestinal system (16.2%), followed by the cardiovascular system (11.2%), and occasionally in the neuropsychiatric domain (17%) [75]. The gastrointestinal disorders that have been documented during rivastigmine therapy include diarrhea, nausea, vomiting, and weight loss [20]. These outcomes are attributable to substantial and prevalent fluctuations in plasma concentrations of the pharmaceutical agent [72]. Less prevalent but noteworthy are cardiac disorders, such as bradycardia or QT interval prolongation, which disrupt the normal electrical activity of the heart [76]. Psychiatric symptoms are characterized by insomnia, anorexia, confusion, anxiety, depression, nervousness, increased aggression, hallucinations, and nightmares [77]. Furthermore, the potential for adverse effects, including headaches, dizziness, and syncope, should not be overlooked [30]. Some observations suggest that combination therapy with donepezil demonstrated enhanced efficacy in improving cognitive function when compared with monotherapy involving rivastigmine [48].

### 3.4. Galantamine

#### 3.4.1. Characteristics of Galantamine

**Figure 5:**
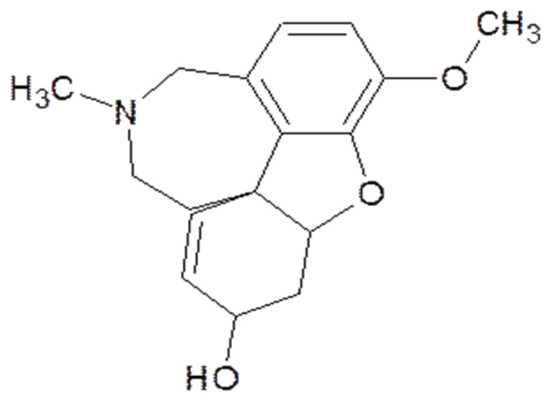
Structural formulas of galantamine (prepared using ChemSketch).

Galantamine ((4aS,6R,8aS)-5,6,9,10,11,12-Hexahydro-3-methoxy-11-methyl-4aH-[1] benzofuro [3a,3,2-ef] [2]benzazepin-6-ol) is a heterocyclic phenanthridine derivative (Figure 5) that was isolated from the bulbs and the flowers of *Galanthus woronowii*. This alkaloid belongs to the Amaryllidaceae family, wherein the biosynthetic pathway is produced from 4′-o-methyl norbelladine [54]. This family of plants contain several diverse structural types, such as some differences in intramolecular oxidative couplings: ortho-para’, para-para’, and para-ortho’. Galantamine belongs to “para-ortho”, which is why this coupling provides galantamine’s pharmacological features [78].

**Figure 6:**
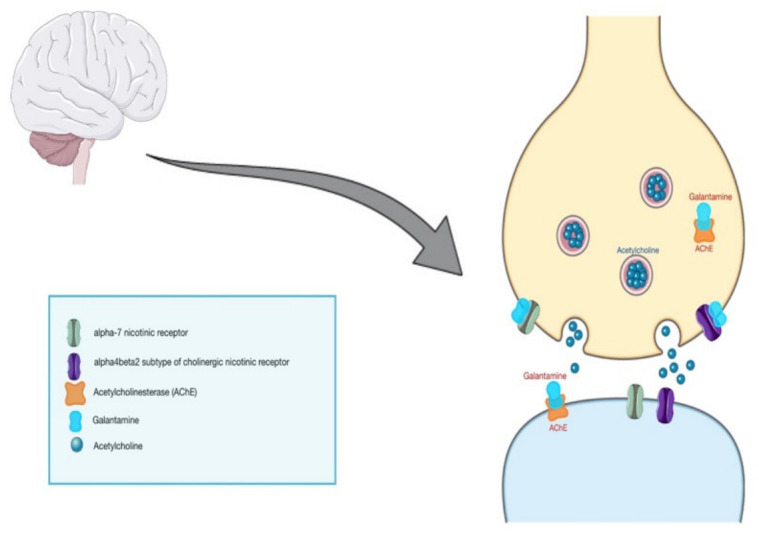
Molecular mechanism of action of galantamine. Galantamine selectively inhibits AChE in the CNS, as a result increasing the ACh level in synaptic fissure. In addition, it is a positive allosteric modulator at presynaptic alpha-7 nicotinic receptors and the alpha4beta2 subtype of cholinergic nicotinic receptors. Adapted from Servier Medical Art (https://smart.servier.com [accessed on 28 May 2026]), licensed under CC BY 4.0 (https://creativecommons.org/licenses/by/4.0/).

The interesting galantamine biological activity combined with its limited availability from natural sources has increased the interest in approaches to its total synthesis. One of the key features that makes galantamine suitable for the treatment of AD is its selective inhibition of AChE in the CNS, with minimal effects on peripheral tissues (Figure 6). Its notable biological activity, combined with its limited availability from natural sources, has significantly increased interest in developing efficient approaches to its total synthesis [54].

In addition, it has been demonstrated to function as a positive allosteric modulator at presynaptic alpha-7 nicotinic receptors, as well as the alpha4beta2 subtype of cholinergic nicotinic receptors. The latter constitute the most abundant nicotinic receptor in the brain and have been shown to enhance nicotinic neurotransmission [79]. The administration of galantamine has been demonstrated to improve cognitive function and memory in patients diagnosed with AD [80]. Recent studies have begun to demonstrate that galantamine has the ability to stimulate neural progenitor cells in the subgranular zone through activation of the M1 muscarinic receptor [81]. Furthermore, galantamine has been observed to promote the survival of newly formed cells in the granular cell layer through activation of the alpha-7 receptor. It has been suggested that insulin-like growth factor 2 (IGF-2) plays a role in the effect of galantamine, as evidenced by the survival of immature cells in the granule cell layer that are two weeks old [54].

#### 3.4.2. Pharmacokinetics of Galantamine

Galantamine has been shown to have a linear pharmacokinetic profile at doses ranging from 8 mg/d to 32 mg/d. The administration of galantamine for the treatment of AD is typically via oral routes. With such administration, the maximum concentration is achieved within an hour. It has been shown that galantamine does not affect the ANS when taken with food. The only thing that decreases is Cmax, while Tmax is delayed by 1.5 h [82,83]. However, it has been demonstrated that this can enhance the glutamatergic system, which can result in neuroprotective effects and an increase in pharmacological potential. Furthermore, the substance exhibits cholinesterase inhibition, which suggests the potential for selective antagonism of NMDA receptors [54]. The result of this process is that the substance exhibits enhanced pharmacokinetic properties, consequently leading to augmented penetration into the brain [84]. With regard to the administration of galantamine, the optimal efficacy of this drug is observed in mild to moderate forms of AD. Therefore, the optimal time for administration of the drug is at this particular time. When distributing galantamine, the volume is 175 L. The ability of galantamine to bind to plasma proteins is 18%. A total of 52.5% of galantamine in whole blood is distributed to erythrocytes. Its concentration in blood is 1.2 times higher than in plasma [83,85]. Galantamine is metabolized by cytochrome P450 (CYP2D6 and CYP3A4) [79]. It is also a drug that exhibits anti-apoptotic activity as a scavenger receptor of ROS [79]. Thanks to its ability to cross the blood–brain barrier, galantamine has been investigated for neurological conditions, giving the drug historical significance beyond its current use in the treatment of AD [86].

#### 3.4.3. Efficacy and Safety

Galantamine has been demonstrated to exert an influence on nicotinic receptors, thereby inducing an augmentation in postsynaptic conduction. This phenomenon possesses the potential to exert a substantial impact on patients in the early stages of AD [87]. Moreover, the administration of the drug Nivalin in conjunction with galantamine has been observed to induce a state of muscle relaxation during surgical procedures [88]. Studies have demonstrated that the bioavailability of this alkaloid to the brain is suboptimal following oral administration. The aforementioned route has been demonstrated to induce adverse effects, predominantly of a gastrointestinal nature, including vomiting, diarrhea, and abdominal discomfort [54,89]. Despite the occurrence of such side effects, methods have been developed to circumvent these issues through intranasal administration in aerosol form. When orally administered galantamine, there was no change in laboratory blood tests or in the ECG. Gln-1062, an intranasal prodrug of galantamine, was reported to cause mild nasal irritation, including dryness and redness of the nasal mucosa in some study participants [90]. Table 1 provides the characteristics of galantamine.

## 4. Analysis of Clinical Efficacy of AChEI’s in Alzheimer’s Disease—Analysis of Cohort Studies

### 4.1. Donepezil

**Figure 7:**
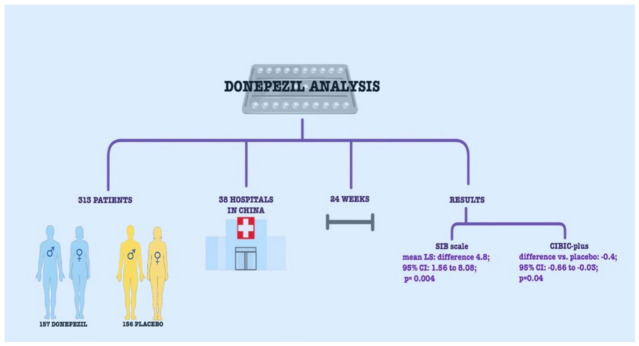
Visualization of the donepezil analysis, multicenter, double-blind, randomized study conducted in 38 hospitals in China. The study group consisted of 313 patients: 157 patients taking donepezil and 156 in the placebo group. The clinical study lasted 24 weeks, resulting in a marked improvement in scores on the SIB scale and CIBIC-plus in the group receiving donepezil. Adapted from Image(s) provided by Servier Medical Art (https://smart.servier.com) [accessed on 28 May 2026], licensed under CC BY 4.0 (https://creativecommons.org/licenses/by/4.0/).

In the context of the donepezil analysis, a 24-week, multicenter, double-blind, randomized study with a placebo group, conducted in a parallel setting, has been described. The study population comprised 313 patients from 38 hospitals in China, with 157 patients allocated to the donepezil group and 156 patients to the placebo group. The group receiving donepezil demonstrated a marked improvement in scores on the SIB scale (mean LS: difference 4.8; 95% CI: 1.56 to 8.08; *p* = 0.004) and CIBIC-plus (difference vs. placebo: −0.4; 95% CI: −0.66 to −0.03; *p* = 0.04) after 24 weeks. The utilization of donepezil over a period of 24 weeks demonstrated superior efficacy in comparison to the placebo, exhibiting both satisfactory tolerability and safety in Chinese patients afflicted with AD. The findings of the study corroborate the legitimacy of administering the pharmaceutical agent to patients afflicted with advanced stages of the disease within the Chinese demographic (see Figure 7) [91].

In another study regarding donepezil, a total of 30 studies comprising 8257 participants were included in the review. Of these, 28 provided sufficient data for meta-analysis. A total of international studies were conducted, including patients from Canada, the European Union, and the United States. The majority of the studies had a duration of six months or less, with one small study extending for 52 weeks. The donepezil capsules were primarily administered at doses of 5 mg or 10 mg per day during the course of these studies. Two studies incorporated a slow-release oral formulation containing 23 mg/day. The primary analysis compared the efficacy and safety of the 10 mg/day dose to the placebo after 24 to 26 weeks of treatment. The analysis encompassed data from 13 studies, comprising a total of 3396 participants. The administration of donepezil has been associated with substantial improvements in several key domains. These include cognitive function, as measured by the ADAS-Cog (mean difference [MD] −2.67); the MMSE (MD 1.05); the SIB (MD 5.92); daily functioning (ADCS-ADL-sev: MD 1.03); and clinician-assessed global improvement (OR 1.92). The study revealed that there were no significant differences observed between donepezil and the placebo with respect to behavioral symptoms, as measured by the Neuropsychiatric Inventory (NPI), or quality of life, as assessed by the Quality of Life (QoL) scale. A higher percentage of patients taking donepezil discontinued treatment or experienced adverse effects (72% vs. 65%). No disparities in healthcare resource utilization were observed. In a dose comparison, a daily dosage of 5 mg led to a reduction in the incidence of adverse effects; however, it yielded only modest enhancements in cognitive function. The 23 mg dosage did not demonstrate a superiority in efficacy in comparison to the 10 mg dosage; however, it elicited a higher incidence of adverse effects. A moderate degree of evidence suggests that donepezil may offer limited benefits in terms of cognitive function, daily living, and global clinical assessment in patients diagnosed with mild, moderate, or severe Alzheimer-type dementia who are undergoing treatment for a period of 12 to 24 weeks [92].

In the British study, 295 participants diagnosed with moderate to severe AD (a score of 5 to 13 on the standardized MMSE, in which higher scores indicate better cognitive function) and who had been treated with donepezil for at least three months were randomly assigned to one of four groups: continuation of donepezil therapy, discontinuation of donepezil, discontinuation of donepezil and initiation of memantine therapy, or continuation of donepezil with the simultaneous inclusion of memantine. The duration of treatment in the study was 52 weeks. The two endpoints included an assessment of cognitive function using the MMSE test and an assessment of functional ability on the Bristol Activities of Daily Living Scale (BADLS), with higher scores indicating a greater degree of disability (range 0–60). The minimally clinically significant changes were 1.4 points for the MMSE and 3.5 points for the Barthel ADLs, respectively. In patients diagnosed with moderate to severe AD, ongoing treatment with donepezil has been associated with sustained cognitive benefits that surpass the threshold for clinical significance. Additionally, significant improvements in functioning have been observed over a 12-month period of follow-up [60].

### 4.2. Rivastigmine

An analysis of a prospective, randomized, multicenter, double-blind, placebo-controlled study with parallel comparison groups was conducted for rivastigmine. The study encompassed 725 patients from 45 medical centers across Europe and North America, diagnosed with mild to moderately severe probable AD. At the conclusion of the study, cognitive deterioration was observed in patients in the placebo group. Patients administered a higher dosage of rivastigmine demonstrated enhanced Alzheimer’s Disease Assessment Scale-Cognitive Section (ADAS-Cog) scores in comparison to the placebo group (*p* < 0.05). A greater proportion of patients in the higher-dose group demonstrated improvement by at least four points, in contrast to the placebo group, which exhibited a lower percentage of patients with such improvements. (24% [57/242] vs. 16% [39/238]). A global function assessment, as determined by clinicians based on patient histories, revealed a substantial enhancement in the higher-dose cohort in comparison with the placebo group (*p* < 0.001). Furthermore, a greater proportion of patients demonstrated improvement in the higher-dose group (37% [80/219] vs. 20% [46/230]). The mean scores on the Progressive Functional Deterioration Scale demonstrated an improvement in the higher-dose group, while the placebo group exhibited a decline. Rivastigmine has been demonstrated to be both effective and well-tolerated. The treatment proves to enhance cognitive function, facilitate participation in daily activities, and improve overall clinical scores in patients diagnosed with mild to moderately severe AD [93].

In a subsequent investigative study characterized by its open-access approach, observational nature, multicenter design, and non-randomized methodology, a sample of 217 patients from Sweden was included in the analysis. The treatment regimen involving rivastigmine was initiated for 62% of the subjects (n = 135), with 24-month follow-up data indicating that 62% of these patients had continued their treatment for a period of 24 months. The primary reasons for discontinuation of the treatment included the necessity for institutional care and the occurrence of adverse effects. Among those continuing treatment, 80% (MMSE) and 67% (ADAS-Cog) demonstrated a decline in cognitive deterioration (≤4 points). A total of 44 percent of patients demonstrated no change or improvement in their CIBIC scores. Administration of rivastigmine resulted in stabilization of the patients, in some cases delaying the onset of further deterioration in their symptoms [94].

Another study was a post hoc analysis of a 24-week, prospective, international, randomized, double-blind, placebo-controlled, active-drug study involving 892 patients. The study, characterized as international, did not specify the country of origin of the patients. However, the involvement of the St. Louis University School of Medicine indicates that the majority of the participants were from the United States. The patients were administered patches (9.5 milligrams every 24 h), capsules (6 milligrams twice daily), or the placebo. After 24 weeks, both forms of rivastigmine demonstrated improvements in overall scores on the ADCS-ADL (patches: *p* = 0.013; capsules: *p* = 0.039). The utilization of capsules was found to be superior for basic activities of daily living (ADLs) (*p* = 0.012). For more complex activities, the efficacy of slices was demonstrated (*p* = 0.056). In terms of autonomy, slices were also found to be advantageous (*p* = 0.017). Rivastigmine, administered in both its conventional and modified forms, has been reported to enhance daily functioning in patients diagnosed with probable AD [95].

### 4.3. Galantamine

A multinational clinical trial was conducted over the course of six months and included 653 patients from various countries, including Finland, France, the Netherlands, Canada, Germany, Norway, Sweden, and the United Kingdom. The patients presented with mild to moderate AD. The trial was designed as a six-month, clinical, parallel-group, double-blind, placebo-controlled analysis. Following a six-month period of treatment, subjects who received galantamine demonstrated a marked improvement in cognitive function, as evidenced by significant increases in scores on the 11-item cognitive subscale of the AD Assessment Scale (ADAS-Cog) when compared to those who received a placebo. The higher dose of galantamine was associated with a significant increase in ITT analysis outcomes (*p* < 0.001 for both doses). In addition, galantamine demonstrated superior efficacy in comparison to the placebo, as indicated by the CIBIC-plus global assessment (*p* < 0.05). Notably, the higher dose of galantamine exhibited a substantial impact on the Dementia Disability Rating Scale, with a mean treatment effect of 3.4 points and a *p*-value of <0.05. Galantamine exhibits efficacy and good tolerability in the treatment of AD [96].

Another study that was considered in the context of galantamine—which was also international in scope—was a randomized, double-blind, placebo-controlled trial. The study was conducted at 43 research centers in the U.S., Canada, the United Kingdom, South Africa, Australia, and New Zealand. A total of 386 patients with probable AD (MMSE: 11–24, ADAS-Cog ≥12) were randomly assigned to receive either a placebo or galantamine (24 or 32 mg/day). After a period of three months, patients treated with galantamine exhibited significantly superior scores in cognitive function (a difference of 1.9 points in ADAS-Cog, *p* = 0.002) and global CIBIC-plus score (21% vs. 37% in the placebo group, *p* < 0.001). Galantamine demonstrated efficacy in enhancing both fundamental and complex activities of daily living (ADLs). No substantial alterations were observed in the behavioral symptoms. The adverse effects (predominantly gastrointestinal) were classified as mild to moderate. A study of patients who received higher doses revealed that more than 80% of them completed the study. The administration of galantamine in a flexible manner was found to be well-tolerated by the subjects and yielded cognitive and functional benefits [97].

A further analysis was conducted on a study comprising 978 patients from the United States. The study design entailed a double-blind, placebo-controlled, multicenter research approach, spanning a duration of five months. The study encompassed a total of 978 patients residing in the United States. Following a 4-week preliminary period, patients were randomly assigned to the placebo or galantamine (8, 16, or 24 mg/day). After a period of five months, galantamine administration at doses of 16 and 24 milligrams per day resulted in substantial enhancements in the ADAS-Cog (3.3 and 3.6 points, respectively, *p* < 0.001), CIBIC-plus global assessment, activities of daily living, and behavioral symptoms. The dropout rate due to side effects was low (6–10%) and similar to the placebo (7%). The most common adverse effects were mild, primarily of a gastrointestinal nature. Interestingly, this study demonstrated that slow-dose escalation significantly enhanced patient tolerance for treatment [98]. Clinical efficacy of AChEIs in Alzheimer’s disease are summarized in Table 2.

## 5. Plant Extracts

Supposedly at present, there are no drugs that are capable of effectively slowing the progression of AD. A plethora of synthetic formulations, including cholinesterase inhibitors and NMDA receptor antagonists such as donepezil, rivastigmine, galantamine and memantine, are currently available on the market and have been shown to provide symptomatic relief. The majority of therapeutic interventions are centered on the alleviation of symptoms, with a particular emphasis on the initial phases of the disease. Therapies developed to date for the treatment of AD have not been entirely efficacious, and long-term exposure to synthetic drugs often leads to serious side effects. Consequently, the utilization of herbal therapies has emerged as a preferred alternative to conventional treatment regimens, a trend that has prompted a surge in research endeavors in recent years. A plethora of herbs, including Ginkgo biloba, Bacopa monnieri, Withania somnifera, and Curcuma longa, have already been employed to alleviate the symptoms of certain neurological disorders. The therapeutic efficacy of these botanical species is attributed to a diverse profile of secondary metabolites, including flavonoids, terpenoids, polyphenols (such as curcuminoids), saponins, and various alkaloids. Operating synergistically, these distinct classes of bioactive compounds exhibit potent antioxidant properties, mitigate amyloid aggregation, and significantly inhibit AChE activity. Furthermore, these phytochemicals modulate neuroinflammatory pathways and regulate key proteins implicated in the pathogenesis of AD, thereby establishing a comprehensive framework for neuroprotection and cognitive support. There is currently a global trend toward the wider therapeutic inclusion and recognition of herbal medicines. These have a long history of use in the treatment of certain brain disorders, including those intrinsically linked to AD in some parts of the world. In comparison with synthetic drugs, herbal medicines are distinguished by their high long-term tolerability and low incidence of adverse effects, such as sleep disturbances, withdrawal symptoms, or toxic effects on other vital organs. These adverse effects are common drawbacks of synthetic alternatives. The present research endeavors to concentrate on the development of effective diagnostic methods and the identification of new pharmaceutical agents. The potential for the discovery of new drugs derived from plant extracts is significant [99,100,101].

### 5.1. Huperzine A

Huperzine A (HupA) is an alkaloid that was first identified from *Huperzia serrata* by Chinese scientists in the 1980s. and is employed in traditional Chinese medicine for the treatment of dementia [102]. The mechanism of action of this alkaloid is believed to be through inhibition of the cholinergic enzyme AChE. It was the first drug of its kind to be clinically approved in China for the treatment of AD, and this approval was granted in 1996. The biological functions of HupA have been the subject of study both in vitro and in vivo, and its role in neuroprotection renders it a promising candidate as a drug to treat AD [103,104].

Gut et al. [105] conducted a clinical trial in Pakistan involving 100 participants, including 50 patients diagnosed with AD and 50 healthy individuals as a control group, selected according to specific inclusion criteria. Patients were recruited from Civil and BV medical facilities in Bahawalpur and Nishtar in Multan. The study was an eight-week, double-blind, clinical analysis designed to evaluate the therapeutic effect on neurological functions. Following the conclusion of the full course of treatment, those administered HupA exhibited a substantial and quantifiable enhancement in overall cognitive function, as determined by the Addenbrooke’s Cognitive Examination (ACE-III) test. This enhancement was accompanied by a statistically significant increase in test scores when compared to the baseline values recorded prior to the commencement of treatment. ANOVA statistical analysis demonstrated that the therapeutic intervention resulted in a significant enhancement in cognitive performance and key neuropsychological parameters in all patients diagnosed with AD. As demonstrated by the results of the Trail Making Test (TMT), HupA has also been shown to be highly efficacious in reducing visual attention deficits, task switching processes, and cognitive flexibility. A substantial enhancement in the functionality of the neural networks responsible for decision-making and executive processes was observed. ANOVA measurements also confirmed a reduction in reaction time in patients and an exceptionally favorable tolerance profile of the therapy itself, which has a direct impact on optimizing the daily functioning of patients in the pathological stage of AD [105].

Rafii et al. [106] conducted a multicenter, double-blind, placebo-controlled phase II clinical trial involving 210 patients over the age of 50 diagnosed with probable AD. Participants were randomly assigned to three study groups: those receiving a maintenance dose of 200 μg of HupA, a progressive dose increasing to 400 μg, and a placebo control group. The efficacy of the intervention was assessed using standardized tools. The following instruments were employed for the purpose of assessment: the ADAS-Cog, the MMSE, the ADCS-ADL, and the CGIC global clinical status scale. A thorough examination of the findings revealed that the administration of a dosage of 200 μg, given twice daily, did not result in a statistically significant enhancement in cognitive function when compared to the placebo. In the group administered 400 μg, a transient yet significant enhancement in the ADAS-Cog scale was observed at week 11. However, at week 16, this effect lost its statistical significance. Furthermore, HupA demonstrated no superior efficacy in domains of activities of daily living (ADL) and overall clinical condition (CGIC). Despite the absence of substantiated evidence regarding the efficacy of the drug in patients with mild to moderate disease, the study demonstrated a favorable safety profile. The substance was well-tolerated by the participants, and no serious adverse events directly related to the administration of the drug were reported during the experiment [106].

### 5.2. Posiphen

Posiphen, identified as a non-natural enantiomer of phenserine with a 3aR configuration, is a compound for which the first practical total synthesis was reported in 1997. It is a non-natural analogue of physostigmine, an alkaloid that is found primarily in the Calabar bean (*Physostigma venenosum*). In the context of its potential therapeutic use in AD, posiphen exhibits significantly lower inhibitory activity against AChE and BChE compared to phenserine. Nevertheless, preclinical studies conducted in animal models and cell cultures have confirmed its ability to reduce inflammatory responses, albeit to a lesser extent than phenserine, as well as its neuroprotective effects. It is evident that clinical trials are currently underway to investigate the potential utilization of this compound in the treatment of AD [107,108].

An international randomized clinical trial was conducted over the course of several weeks and with a total of 120 participants. Subsequent to this, a non-randomized clinical trial was conducted among patients diagnosed with MCI. The study was designed as a clinical analysis using ascending doses (SAD and MAD), conducted as a double-blind, placebo-controlled trial based on single and multiple ascending dose phases. Following a ten-day treatment period, patients receiving posiphen demonstrated a significant enhancement in their amyloidogenic biomarker profile. This phenomenon was confirmed by statistically significant decreases in sAPPα and sAPPβ concentrations in cerebrospinal fluid when compared to the placebo group. A higher dose of the drug was associated with an additional, significant reduction in tau and p-tau protein levels (*p* < 0.05 for all doses used). Furthermore, analysis of amyloid Aβ42 levels demonstrated the drug’s superior efficacy in comparison to the placebo, with maximum doses exhibiting the most substantial impact on sAPPβ levels. Posiphen was found to have both a satisfactory biological efficacy and a favorable safety profile in the treatment of AD. The only adverse events were classified as mild or moderate, with none classified as severe [109].

A double-blind, randomized, placebo-controlled, ascending-dose clinical trial was conducted at five centers in the United States, in accordance with a protocol that had been approved by the IRB. The trial lasted 21–23 days and involved 40 patients. The study comprised patients diagnosed with MCI or mild dementia resulting from AD. The analysis was based on the administration of increasing doses of the drug, ranging from 60 mg, 120 mg, and 180 mg per day. At the conclusion of the 21-day treatment period, no significant decreases in the concentrations of amyloidogenic biomarkers sAPPα and sAPPβ in cerebrospinal fluid were observed in patients receiving posiphen in comparison to the placebo group. A marginal decline in total tau and p-tau181 protein levels was observed at the highest dose, but further research is required to substantiate these findings. Moreover, despite the absence of statistically significant differences in amyloid synthesis inhibition in standard measurements when compared to the placebo, advanced kinetic modeling suggested a potential relationship between the drug and reduced APP protein production through translation inhibition. It is also noteworthy that the analysis of data from a single patient receiving a dose of 180 mg indicated a possible effect of the drug on reducing the production of precursor proteins. Posiphen was found to be safe and generally well-tolerated, with no significant alterations in the vital signs of the subjects and no complaints related to the substance itself [110].

### 5.3. Phenserine

Phenserine is a phenylcarbamate derivative that exhibits a structure similar to that of rivastigmine and is a selective, non-competitive AChEI. In 1995, phenserine was identified as a potential therapeutic compound for the treatment of AD. Preclinical studies in animal models have validated the drug’s satisfactory safety profile and favorable tolerability. Furthermore, studies conducted in cell cultures indicate a beneficial effect of phenserine on the development of AD [107].

Winblad et al. [111] conducted an international clinical trial that spanned between 12 and 26 weeks and involved 255 patients. The subjects of this study were men and women aged 50 years or older with probable AD. The study was conducted as a randomized clinical analysis with a placebo control group in parallel groups and using a double-blind method. The results obtained after the conclusion of treatment periods lasting more than 12 weeks demonstrated a marked enhancement in cognitive function in patients administered the preparation. A statistically significant change in the 11-point ADAS-Cog scale score was observed in the experimental group when compared to the placebo group. A detailed analysis confirmed that the administration of a higher dose of phenserine was associated with a more significant improvement in cognitive performance in patients receiving long-term treatment. Furthermore, the CIBIC-plus global clinical assessment demonstrated a discernible trend, thus indicating the therapeutic superiority of the pharmaceutical agent. The utilization of the pharmaceutical agent was concomitant with a favorable safety profile and stabilization of clinical indicators. The only adverse effects reported were nausea, vomiting, dizziness, and headaches. The findings of this preliminary investigation substantiate the therapeutic efficacy of phenserine in the management of symptoms associated with mild to moderate AD. However, the data obtained did not allow for an assessment of the substance’s effect on amyloid metabolism or on the modification of the pathogenic mechanisms underlying the progression of AD [111].

Nordberg et al. carried out a clinical trial over a period of 12 months, which included a period of 3 months in a double-blind, randomized, placebo-controlled study. The study was conducted in Sweden at two clinical centers. The study group consisted of 20 patients diagnosed with mild AD. The study subjects fulfilled the criteria for selection by obtaining a score of at least 21 on the MMSE scale. The study was based on the analysis of the levels of phenserine administered for a period of 6 months and 12 months at a dosage of 15 mg twice a day. After the completion of the study period, a statistically significant increase was observed in the levels of biomarkers sAβPPα, sAβPPβ, Aβ40 (*p* < 0.05), and T-tau (*p* < 0.01) in the CSF in patients treated with phenserine compared to the baseline levels. A decrease was observed in the levels of Aβ42 and Aβ42/40. This decrease was observed in patients treated for a longer period compared to those treated for a shorter period. In addition, a significant positive correlation was observed between the increase in the levels of these biomolecules and improvement in regional cerebral glucose metabolism (rCMRglc). The above studies have also shown a marked improvement in cognitive function with specific emphasis on attention and episodic memory. It is pertinent to mention here that the long-term effect of the drug was marked by the stabilization of glucose metabolism in the cerebral cortex. This phenomenon was observed even though the disease process was progressing, which is an indication of the maintenance of synaptic activity.

The safety profile of phenserine was also monitored by observing adverse events, vital signs, and physical examinations, laboratory tests, and ECGs. No adverse effects were observed [112].

## 6. Discussion

A broad array of pharmaceutical agents that function as AChEI is involved in the therapeutic management of AD. These agents are rivastigmine, galantamine, and donepezil, among others. Studies have indicated the moderate to pronounced efficacy of these preparations in treating AD, particularly in the mild and moderate stages. However, it must be emphasized that these interventions are exclusively symptomatic; their capacity to alter the natural history of the disease remains unproven. An analysis of the available data indicates improvements in cognitive, global, and behavioral functions are achieved following the administration of rivastigmine. The studies examined in this publication demonstrated enhancements in scores on three distinct diagnostic scales: the MMSE, the ADAS-Cog, and the Clinical Dementia Rating. These improvements were observed in comparison to the placebo group. A significant proportion of subjects continued treatment, suggesting its relatively good tolerance. In one such study, the efficacy of rivastigmine was also tested and confirmed in the long-term. Another publication compared the effectiveness of the drug according to the form of ingestion. The study demonstrated that in terms of ADL improvement, capsules were the most effective. However, in terms of facilitating the performance of complex activities and autonomy, patches were found to be the most effective [93,94,95]. The existence of a second route of absorption is significant because bypassing the gastrointestinal tract results in the first-pass effect and metabolization in the liver being avoided. A notable property of rivastigmine is its modest capacity to bind to other proteins, which results in minimal interaction with other therapeutic agents. Galantamine has also been observed to improve the aforementioned domains, with outcomes that are distinctly influenced by suitable dosage adjustments. A series of cohort studies demonstrated that the pharmaceutical treatment had a positive impact on cognitive performance, daily functioning, and global assessment. However, no single study observed substantial alterations in behavioral symptoms. The decision to forgo treatment was made by a minority of subjects, which suggests that the medication is well-tolerated [85,86,90]. Notably, the compound exhibits dual effects, including allosteric modulation of nicotinic receptors, which has improved nicotinic neurotransmission within the CNS. This property may hold particular significance in the early stages of AD [80]. Many studies helped to record a stimulatory effect of galantamine on neuronal progenitor cells in the subgranular zone and on the survival of new cells in the granular layer [79]. Nevertheless, the low bioavailability to the brain after oral administration constitutes a significant problem for treatment with this compound, and further research is being conducted. Donepezil demonstrated moderate enhancement in all of the scales previously mentioned. The treatment was established to be ineffective in reducing behavioral symptoms or enhancing quality of life. The presence of an elevated risk of disordered symptoms was also observed. The inclusion of memantine, a non-competitive NMDA receptor antagonist, in the therapeutic regimen resulted in enhanced outcomes [60,92]. However, the positive neuroprotective effects of the substance were noted after observation that the cytotoxic effects of glutamate were lowered, while the AChE isoforms linked to neuroprotection were upregulated. All three substances have been shown to be useful in reducing the symptoms associated with AD, confirming their clinically significant utility in helping people struggling with the disease. Adverse effects were reported for all three drugs, primarily gastrointestinal complaints due to the key role of ACh as a transmitter in the parasympathetic system. These symptoms included abdominal pain, vomiting, and diarrhea [77]. Rivastigmine has also been associated with the occurrence of cardiovascular and neuropsychiatric complications [61]. Current pharmacotherapies for AD primarily provide symptomatic relief and have a limited impact on disease progression. Plant-derived compounds exhibit potentially beneficial biological properties, including antioxidant activity and effects on neurotransmitter systems, suggesting a possible supportive role in therapy. However, available clinical evidence remains limited and requires further validation. This underscores the necessity for a more thorough examination of the potential benefits of these substances. Further analysis of the subject may facilitate the development of a remedy for a disease that is currently among the most significant health problems in modern society.

The introduction of herbal therapies has become an alternative to conventional treatment regimens, which in recent years has provided access to scientific research. Herbal medicines in the published literature have been associated with long-term tolerance and effects such as sleep disturbances, withdrawal symptoms, or side effects on other vital organs [103,104,105,106,107,108,109,110,111]. In the case of the alkaloid HupA, used as an AChEI [50,103,104], patients diagnosed with AD (who received it) showed improved cognitive and neuropsychological parameters after treatment completion in all patients. Improvements were observed in the neural networks responsible for decision-making and executive processes [105]. Although posiphen exhibits significantly weaker inhibitory effects on AChE and BChE compared to phenserine, preclinical studies in animal models and cell cultures have confirmed its ability to reduce inflammatory responses as well as its neuroprotective effects. Clinical trials are currently underway to investigate the potential use of this compound in the treatment of AD [108,109]. These studies are promising, as significant improvements in amyloidogenic biomarker profiles have been observed. Preclinical studies on animal models showed satisfactory safety profile and good tolerability of phenserine. Further research conducted in cell cultures have indicated a beneficial effect of phenserine on the progression of AD [107] and improved cognitive function in patients receiving long-term treatment [111]. Phenserine use was associated with a favorable safety profile and stabilization of clinical parameters. Nordberg et al. [112] observed a statistically significant increase in CSF biomarker levels of sAβPPα, sAβPPβ, Aβ40, and T-tau in patients treated with phenserine compared to baseline levels. The studies also demonstrated improved cognitive function, with particular emphasis on attention and episodic memory. One recent study also suggests that the selective modulation of muscarinic receptor subtypes (especially M1) can be a promising strategy in treating Alzheimer’s disease. Activation of muscarinic M1 receptors have been shown to influence amyloid precursor protein processing and tau-related pathways. These findings indicate that selective targeting may potentially effect important molecular processes. This approach may provide clinical benefits while reducing peripheral cholinergic adverse effects [113].

### Limitations

A notable finding was the variation in individual outcomes observed across the analyzed research. Differences in efficacy between rivastigmine, donepezil, and galantamine should be interpreted with caution. The presented review is a structured narrative review and because of that, no formal review protocol, PRISMA flow diagram, meta-analysis, or comprehensive risk-of-bias assessment was performed for the chosen studies. The review is not trying to prove one substance’s superiority over another, but to summarize and interpret gathered evidence. The underlying causes of these discrepancies are multifaceted and require further investigation. The first is the variation in the duration of follow-up, which ranged from a minimum of three months to a maximum of 24 months [60,88]. The second possible reason relates to the issue of the nature of the studies, as there have been some with no randomization and no control group [88]. A critical component of this analysis pertains to the selection of the study population. The stage of the disease, age, cohort size, and the presence of other conditions have a very significant impact on the outcome. It is important to acknowledge the inherent challenges associated with accurate stage assessment, particularly in the context of this condition, given its unique characteristics [95,96]. The drugs demonstrated diminished efficacy in advanced stages of AD, where significant neural tissue degeneration had already occurred. However, there is a lack of studies that are sufficiently reliable to determine the effects of the long-term use of drugs in this group on patients’ functioning and the arrest of AD progression.

## 7. Conclusions

AD is a neurodegenerative disorder that affects millions of people worldwide. The exact etiology of AD remains unclear; however, it is known that patients with AD exhibit impairment of central cholinergic neurons. AChE plays a crucial role in neurodegenerative diseases, and understanding its function may contribute to a better comprehension of the pathogenesis and pathophysiology of these disorders. This enzyme performs several important functions common to most of the described conditions, including involvement in oxidative stress and inflammatory responses, participation in apoptosis, and facilitation of pathological protein adhesion. AChE is an enzyme responsible for degrading one of the most important neurotransmitters. Disturbances in its levels may contribute not only to neurodegenerative diseases, but also to depressive disorders. In addition to enzyme inhibition, currently available AChEIs possess a range of other properties that may help slow disease progression. They act primarily symptomatically rather than causally. However, this review does not fully confirm this theory, as some of these agents may have potential applications in causal treatment. Currently, very few drugs are available for the treatment of AD. Most available medications inhibit cholinesterase, the enzyme responsible for breaking down acetylcholine into choline and acetic acid. Inhibition of cholinesterase results in increased levels of acetylcholine. Well-known cholinesterase inhibitors used in the treatment of AD include donepezil, rivastigmine, and galantamine. However, the precise efficacy and safety of acetylcholinesterase inhibitors in the treatment of AD remain unclear. Studies demonstrate the efficacy of these pharmaceutical agents. However, their effectiveness is limited to the treatment of symptoms. There is currently no clinical evidence to support their efficacy in preventing the development of the condition. Although the results varied between medications, each showed noticeable improvements in AD symptoms. In addition, a few substances such as HupA, posiphen, and phenserine have shown promising results in improving cognitive function and modulating amyloid-related biomarkers, suggesting potential therapeutic benefits beyond currently approved cholinesterase inhibitors. These findings suggest the necessity of conducting further research for additional solutions. This is of untold importance, considering the epidemiological characteristics of the condition, as well as its status as a civilization disease. Although currently available therapies primarily rely on synthetic drugs to manage symptoms of AD, ongoing research is increasingly focused on developing plant-based alternatives. These approaches may offer advantages such as better long-term tolerability and reduced risk of adverse side effects, making them a promising direction for future therapeutic development.

## Figures and Tables

**Table 1:** Characteristics of AChEIs used in the symptomatic treatment of Alzheimer’s disease.

	Donepezil	Rivastagmine	Galantamine
**Route of administration**	Oral	Oral, transdermal	Oral
**Dose ranges [mg/day]**	5–30	Oral: 1.5–6, transdermal: 4.6–17.4	8–32
**Time to maximum concentration [h]**	4	0.8–167 (delayed by food)	1
**Half-life**	70	2	7
**Mechanism of action**	Binding to anionic site in a central location of AChE	Dual inhibition of AChE and BChE	Selective AChEI in the CNS and a positive allosteric modulator of α7 and α4β2 nicotinic receptor
**Metabolism**	Liver—cytochrome P450 isoenzymes 3A4 and 2D6	Cholinesterase	Liver—CYP2D6 and CYP3A4
**Route of elimination**	Renal	Renal	Renal
**Clinical indications**	Treatment of mild to moderate AD	Stabilization of activities of daily living	Clinical benefit in mild-to-moderate stages of the disease
**Efficacy**	Improvement in patients’ cognitive function, activity, and social behavior, the treatment well-tolerated, even despite high levels of comorbidities.	Significant improvements in AD Assessment Scale-Cognitive Subscale	Improvement of cognitive function and memory
**Adverse effects**	Gastrointestinal events, typically nausea, vomiting, diarrhea, constipation, headache, dizziness, and sleep disturbances	Nausea, vomiting, diarrhea, weight loss, insomnia, anxiety, aggression, hallucinations. Cardiovascular, bradycardia and QT interval prolongation	Gastrointestinal disturbances, including nausea, vomiting, diarrhea, and abdominal discomfort

**Table 2:** Clinical efficacy of AChEIs in Alzheimer’s disease.

Analysis Drugs	Patients	Treatment/Doses [mg/Day]	Duration	Results	References
Donepezil	313 patients with symptoms of severe AD	5 for 6 weeks, then 10/day for 18 weeks	24 weeks	A marked improvement in scores on the SIB scale (mean LS: difference 4.8; 95% CI: 1.56 to 8.08; *p* = 0.004) and CIBIC-plus (difference vs. placebo: −0.4; 95% CI: −0.66 to −0.03; *p* = 0.04) after 24 weeks.	[91]
	8257 participants (meta-analysis)	Mostly: 5 or 10, one 23	≤6 months	Substantial improvements in several key domains (cognitive function, daily functioning, clinician-assessed global improvement).	[92]
	295 participants	Donepezil: 10 + placebo memantine, Donepezil: 5 during weeks 1–4 and placebo donepezil starting in week 5, plus placebo memantine, Donepezil at a dose of 5 during weeks 1–4, with placebo donepezil starting in week 5, plus memantine:5 in week 1, with the dose increased in 5-mg increments weekly to a dose of 20 from week 4 on, Donepezil: 10 and memantine at a dose of 5 in week 1, with the dose increased in 5-mg increments weekly to a dose of 20 from week 4 on.	52 weeks	Minimally clinically significant changes: 1.4 points for the MMSE and 3.5 points for the Barthel ADLs.	[60]
Rivastigmine	725 patients	Placebo, 1–4 mg/day, 6–12 mg/day.	26 weeks	Rivastigmine has been demonstrated to be both effective and well-tolerated. The treatment has been demonstrated to enhance cognitive function, facilitate participation in daily activities, and improve the overall clinical scores in patients diagnosed with mild to moderately severe AD.	[93]
	217 patients		24 months	Administration of rivastigmine resulted in stabilization of patients, in some cases delaying the onset of further deterioration in their symptoms.	[94]
	892 patients	Placebo, 9.5 mg/day (patches), 6 mg twice a day (capsules).	24 weeks	Rivastigmine has been demonstrated to enhance daily functioning in patients diagnosed with probable AD.	[95]
Galantamine	653 patients of whom 80% (525) completed the study.	Placebo, 24 mg/day, 32 mg/day.	6 months	Galantamine has demonstrated efficacy and good tolerability in the treatment of AD, patients in the higher dose galantamine group had significantly better scores on the disability assessment for dementia scale than patients in the placebo group.	[96]
	386 patients	Placebo, 24 mg/day, 32 mg/day.	3 months	Galantamine demonstrated efficacy in enhancing both fundamental and complex activities of daily living (ADLs). No substantial alterations were observed in the behavioral symptoms. The adverse effects (predominantly gastrointestinal) were classified as mild to moderate.	[97]
	978 patients	Placebo, 8 mg/day, 16 mg/day, 24 mg/day.	5 months	Galantamine administration at doses of 16 and 24 milligrams per day resulted in substantial enhancements in the ADAS-Cog, CIBIC-plus global assessment, activities of daily living, and behavioral symptoms. The dropout rate due to side effects was low (6–10%) and similar to placebo (7%). The most common adverse effects were mild, primarily of a gastrointestinal nature. Interestingly, this study demonstrated that slow-dose escalation significantly enhances patient tolerance for treatment.	[98]

## Data Availability

No new data were created or analyzed in this study. Data sharing is not applicable to this article.

## Abbreviations

The following abbreviations are used in this manuscript:

AD	Alzheimer’s disease
AChE	Acetylcholinesterase
nAChER	Nicotinic receptors
AChEIs	Acetylcholinesterase inhibitors
ACh	Acetylcholine
CNS	Central nervous system
PNS	Peripheral nervous system
ChAT	Choline acetyltransferase
MMSE	Mini-Mental State Examination test
ADAS-Cog	Alzheimer’s Disease Assessment Scale-Cognitive Section
Cmax	Maximum plasma concentration
BChE	Butyrylcholinesterase
BChEIs	Butyrylcholinesterase inhibitors
Tmax	Time to maximum concentration
MCI	Mild cognitive impairment
MAO-A	Monoaminoxidase A
MAO-B	Monoaminoxidase B
NMDA	N-methyl-D-aspartate
HupA	Huperzine A
